# Identification of m6A- and ferroptosis-related lncRNA signature for predicting immune efficacy in hepatocellular carcinoma

**DOI:** 10.3389/fimmu.2022.914977

**Published:** 2022-08-11

**Authors:** Hongjun Xie, Muqi Shi, Yifei Liu, Changhong Cheng, Lining Song, Zihan Ding, Huanzhi Jin, Xiaohong Cui, Yan Wang, Dengfu Yao, Peng Wang, Min Yao, Haijian Zhang

**Affiliations:** ^1^ Research Center of Clinical Medicine, Affiliated Hospital of Nantong University, and Medical School of Nantong University, Nantong, China; ^2^ Department of Pathology, Affiliated Hospital of Nantong University, Nantong, China; ^3^ Department of Clinical Laboratory, People’s Hospital of Ganyu District, Lianyungang, China; ^4^ Department of General Surgery, Shanghai Electric Power Hospital, Shanghai, China; ^5^ Department of Emergency, Affiliated Hospital of Nantong University, Nantong, China; ^6^ Department of General Surgery, Affiliated Hospital of Nantong University, Nantong, China; ^7^ Department of Immunology, Medical School of Nantong University, Nantong, China

**Keywords:** long noncoding RNA pairs, N6-methyladenosine (m6A) methylation, ferroptosis, hepatocellular carcinoma, immune efficacy

## Abstract

**Background:**

N6-methyladenosine (m6A) methylation and ferroptosis assist long noncoding RNAs (lncRNAs) in promoting immune escape in hepatocellular carcinoma (HCC). However, the predictive value of m6A- and ferroptosis-related lncRNAs (mfrlncRNAs) in terms of immune efficacy remains unknown.

**Method:**

A total of 365 HCC patients with complete data from The Cancer Genome Atlas (TCGA) database were used as the training cohort, and half of them were randomly selected as the validation cohort. A total of 161 HCC patients from the International Cancer Genome Consortium (ICGC) database were used as external validation (ICGC cohort).

**Results:**

We first identified a group of specific lncRNAs associated with both m6A regulators and ferroptosis-related genes and then constructed prognosis-related mfrlncRNA pairs. Based on this, the mfrlncRNA signature was constructed using the least absolute shrinkage and selection operator (LASSO) analysis and Cox regression. Notably, the risk score of patients was proven to be an independent prognostic factor and was better than the TNM stage and tumor grade. Moreover, patients with high-risk scores had lower survival rates, higher infiltration of immunosuppressive cells (macrophages and Tregs), lower infiltration of cytotoxic immune cells (natural killer cells), poorer immune efficacy (both immunophenoscore and score of tumor immune dysfunction and exclusion), higher IC_50_, and enrichment of the induced Treg pathway, which confirmed that the mfrlncRNA signature contributed to survival prediction and risk stratification of patients with HCC.

**Conclusions:**

The mfrlncRNA signature, which has great prognostic value, provides new clues for identifying “cold” and “hot” tumors and might have crucial implications for individualized therapy to improve the survival rate of patients with HCC.

## Introduction

Hepatocellular carcinoma (HCC) is the leading cause of premature death worldwide (1). In 2020, primary liver cancer was the sixth most frequently diagnosed cancer and the third major cancer-related cause of death, with approximately 906,000 new cases and 830,000 deaths (2). Its occurrence is a complex process that involves multiple risk factors (3). Advances have been made in the study of HCC pathogenesis in recent years. For example, viruses, type 2 diabetes, obesity, and alcohol-associated liver disease have been confirmed to be risk factors for HCC (4). Moreover, PD-1/PD-L1 blockade has made great progress in cancer immunotherapy, and it has been proven to dramatically increase the 5-year survival rate of patients with HCC (5). However, the underlying molecular mechanisms of HCC remain largely elusive (6); only 20%–30% of patients generally benefit from PD-1/PD-L1 blockade therapy (7, 8). Therefore, we need to find more prognostic markers to stratify patients with HCC according to the risks and perform individualized therapy to improve the survival rate of patients with HCC.

Long noncoding RNAs (lncRNAs) are one of the main regulatory factors of gene expression (9). The expression of lncRNA not only affects transcription, translation, protein modification, and other mechanisms but also affects the inhibition, treatment, and prognosis of cancer through cellular signaling pathways, playing a key role in tumorigenesis, metastasis, prognosis, and diagnosis (10). Research has shown that the signature constructed by lncRNAs can function as a prognostic biomarker for cancer to improve the survival of patients with cancer (11). It has been shown that lncRNA, a potential biomarker, is important in tumor growth and metastasis (12). Therefore, it is feasible to study the expression of lncRNAs to evaluate the prognosis of HCC (13). Equally important is N6-methyladenosine (m6A), a methylated modification that occurs in RNA and is involved in the cleavage, transportation, stability, and degradation of noncoding RNA (14). It is widely present in the transcriptome and has become a prominent topic in the field of tumorigenesis research (15, 16). The methylation modification of m6A in various tumors and its effects on RNA metabolism provide new ideas and methods for the early diagnosis and treatment of cancer (17). In addition, researchers have found that m6A regulates ferroptosis through the autophagy signaling pathway in hepatic stellate cells, and m6A modification-dependent ferroptosis contributes to the treatment of liver fibrosis (18). As a new form of iron-dependent oxidative cell death, ferroptosis is of great importance and affects cytological changes, such as increased mitochondrial membrane density and cell shrinkage (19, 20), and can influence the development of liver diseases by regulating intracellular iron levels, production of intracellular reactive oxygen species, and lipid peroxides (21). Ferroptosis also has a tumor-suppressive function and can be used for cancer treatment (22). Interestingly, the researchers found that sorafenib, the only approved first-line agent for patients with HCC, induced ferroptosis (23). Therefore, ferroptosis is of great importance in the treatment and prognosis of HCC (24).

Recently, the construction of a prognostic model based on lncRNA expression has received increasing attention. For example, a 6-lncRNA signature was constructed to predict recurrence-free survival, which provided new clinical evidence for the accurate diagnosis and targeted treatment of patients with HCC (25). However, the 6-lncRNA risk score based on the risk model did not show a better prognostic value than some important clinical traits, such as the TNM stage. Therefore, more prognostic markers that are superior to clinical characteristics need to be identified to evaluate the prognosis of HCC. Recent evidence indicates that the combination of the two biomarkers contributes to improving the accuracy of the model (26). Recently, it has been reported that an m6A-related lncRNA signature was identified to predict prognosis and immunotherapy of HCC (27). The results showed that the prognostic value of this model was superior to that of other clinical traits such as TNM stage and tumor grade. However, the receiver operating characteristic (ROC) curve established based on the m6A-related lncRNA signature indicated that the area under the curve (AUC) values at 1, 3, and 5 years were 0.708, 0.635, and 0.611, respectively. As a result, the value of the signature is acceptable; thus, more accurate prognostic models are needed for the prognosis of HCC. Therefore, it is appropriate to combine more biomarkers to construct a prognostic model with better accuracy. Therefore, we aimed to develop an mfrlncRNA signature based on a group of specific lncRNA pairs associated with m6A regulators and ferroptosis-related genes.

In this study, we first identified a group of specific lncRNAs associated with both m6A regulators and ferroptosis-related genes and then constructed prognosis-related mfrlncRNA pairs. Based on this, we constructed the mfrlncRNA signature and divided patients into high- and low-risk groups. Subsequently, we further evaluated the guiding value of the mfrlncRNA signature to immune efficacy, as well as immune infiltration, drug sensitivity, and biological function in the training cohort, validation cohort, and International Cancer Genomics Consortium (ICGC) cohort, respectively.

## Materials and methods

### Data acquisition

RNA-seq transcriptome data (FPKM value) were obtained from The Cancer Genome Atlas (TCGA) database (https://portal.gdc.cancer.gov). A total of 424 data files containing HCC transcriptome data were downloaded. The same applies to patient clinical information, whose data category was set as “clinical” and the data format set as “BCR XML,” containing 377 data files. Transcriptome data were annotated using human gene profiles to obtain a list of mRNAs and lncRNAs. Patients with multiple locus samples had their gene expression replaced by the average of the multiple samples. If the same gene is detected more than twice, the expression value of the corresponding gene is replaced by its average value. Patients who died on the day of surgery were excluded (28, 29). Finally, 50 normal and 365 tumor samples were enrolled in our study. Half of the HCC samples were randomly selected to form the validation cohort, and the prognostic value of the mfrlncRNA signature was validated. A total of 365 HCC patients with complete data were used as the training cohort, and half of them were randomly selected as the validation cohort. Analysis of clinicopathological features of patients with lung adenocarcinoma in training and validation cohorts is shown in **Supplementary Table S1**. There were no statistically significant differences in clinicopathological features between the training and validation cohorts. The average survival time for the training cohort was 2.045 years, with 121 deaths within 5 years. The mean survival time in the validation cohort was 1.995 years, with 55 deaths within 5 years.

The ICGC database provides sequencing results of transcriptional samples from a variety of tumor tissues, including hepatocellular carcinoma. RNAseq data of 161 cases of hepatocellular carcinoma (LICA-FR) were obtained from the ICGC database (https://dcc.icgc.org/releases/current/Projects), and relevant analysis was considered external validation.

### Acquisition of mfrlncRNA pairs

A total of 502 frlncRNAs were obtained according to the correlation analysis of 214 ferroptosis-related genes and 14,080 lncRNAs, with the following parameters: *R* = 0.5, *p* = 0.001. A total of 108 mfrlncRNAs were obtained according to the correlation analysis of 23 m6A regulators and 502 frlncRNAs using the following parameters: *R* = 0.5, *p* = 0.001. A coexpression network was used to visualize the coexpression relationships. A difference analysis was conducted to identify mfrlncRNAs that were differentially expressed between normal and tumor samples with the following thresholds: logFC = 1 and FDR = 0.05. According to the iteration loop, the expression of two mfrlncRNAs in each mfrlncRNA pair was compared to construct a 0-or-1 matrix. Taking a mfrlncRNA pair as an example, the expression level of mfrlncRNA A/mfrlncRNA B was defined as “1” when the expression level of mfrlncRNA A was greater than mfrlncRNA B, otherwise, it was defined as “0.” When the samples with mfrlncRNA pair expression levels of “0” and “1” account for 20%–80% of all samples respectively, the mfrlncRNA pair was retained.

### Construction of mfrlncRNA signature

Survival-related mfrlncRNA pairs were identified using univariate Cox regression analysis. In total, 35 mfrlncRNA pairs were screened using LASSO regression, and 16 optimal mfrlncRNA pairs with the minimum error used to construct the mfrlncRNA signature were identified using Cox regression. Based on the mfrlncRNA signature, the risk score of patients with HCC was evaluated according to the following formula:


$$\text{risk score}=\sum_{k=1}^{n}\text{coef}\left({\text{mfrlncRNA pair}}^{k}\right)*\text{expr}\left({\text{mfrlncRNA pair}}^{k}\right)$$

According to the risk score, a ROC curve was drawn, and the AUC was calculated to check the accuracy of the mfrlncRNA signature. Maximization of the sum of sensitivity and specificity was taken as the optimal cutoff point to divide the high- and low-risk populations. Kaplan–Meier analysis was performed between the high- and low-risk populations to prove that the risk score can be used as an independent clinical prognostic predictor. Subsequently, the risk score was compared with other clinical traits using univariate and multivariate Cox regression analyses.

Since two rare lncRNAs were not detected in the sequencing results of the ICGC database, corresponding mRNA precursors or small nuclear RNAs with the same transcription efficiency were used to replace them, so as to construct the risk model successfully. AC026356.1 [ENSG00000274964, novel transcript, sense intronic to bicaudal D homolog 1 (BICD1)] was replaced by BICD1. Small nucleolar RNA, H/ACA Box 74A (SNORA74A), encoded by the second intron of the SNHG4 (U19H) gene, was used to replace its host gene SNHG4 (30, 31). Subsequently, we calculated the risk scores of 161 patients based on the previous model coefficients and classified these 161 patients into high- and low-risk groups according to risk scores.

### Immune infiltration analysis

Immune infiltration between high- and low-risk populations was analyzed using several algorithms as follows: First, single-sample gene set enrichment analysis (ssGSEA), which is an extension of the GSEA method, was used to indicate the absolute degree of enrichment of the gene set in patients based on transcriptome data (32). Second, the estimation of stromal and immune cells in malignant tumor tissues using expression data (ESTIMATE) was used to assess the purity of tumors according to the analysis of stromal and immune cells (33). Finally, cell-type identification by estimating relative subsets of RNA transcripts (CIBERSORT), based on gene expression data, was used to estimate the abundance of member cell types in a mixed cell population (34).

### Prediction analysis of immune efficacy

Immune efficacy was analyzed using the LIHC module immunotherapy score (http://tcia.at/). Samples with an immune efficacy score of NA were excluded. The immune efficacy score was used to analyze the differences in immune efficacy between the high- and low-risk populations. Immune checkpoints were analyzed to explore the differences in immune efficacy.

### Function analysis

According to the difference analysis, differentially expressed genes were obtained with the thresholds set as follows: logFC = 1, *p* = 0.001. The duplicated genes were averaged, and genes with low content in all the samples were deleted. The functions of distinct genes between high- and low-risk populations were analyzed using Gene Ontology (GO) and Kyoto Encyclopedia of Genes and Genomes (KEGG) analyses. The annotated gene set file selected was c7.all.v7.4.symbols.gmt (immunologic signatures). Pathway enrichment was analyzed according to GSEA with the following threshold: *p* = 0.05.

### Statistical analysis

Statistical analyses were conducted using R software 4.0.4 obtained (www.r-project.org). Survival, LASSO, and functional analyses were performed based on the Kaplan–Meier “survival” package, “glmnet” package, and “enrichplot” package, respectively. With the “rms” package, the nomogram and calibration plot were analyzed. Half inhibitory concentration (IC_50_) was used to represent drug sensitivity, and chemotherapy response prediction was performed using R software with the “pRRophetic” package. Statistical significance was set at *p* < 0.05.

## Result

### Construction of mfrlncRNA signature

**Figure 1:**
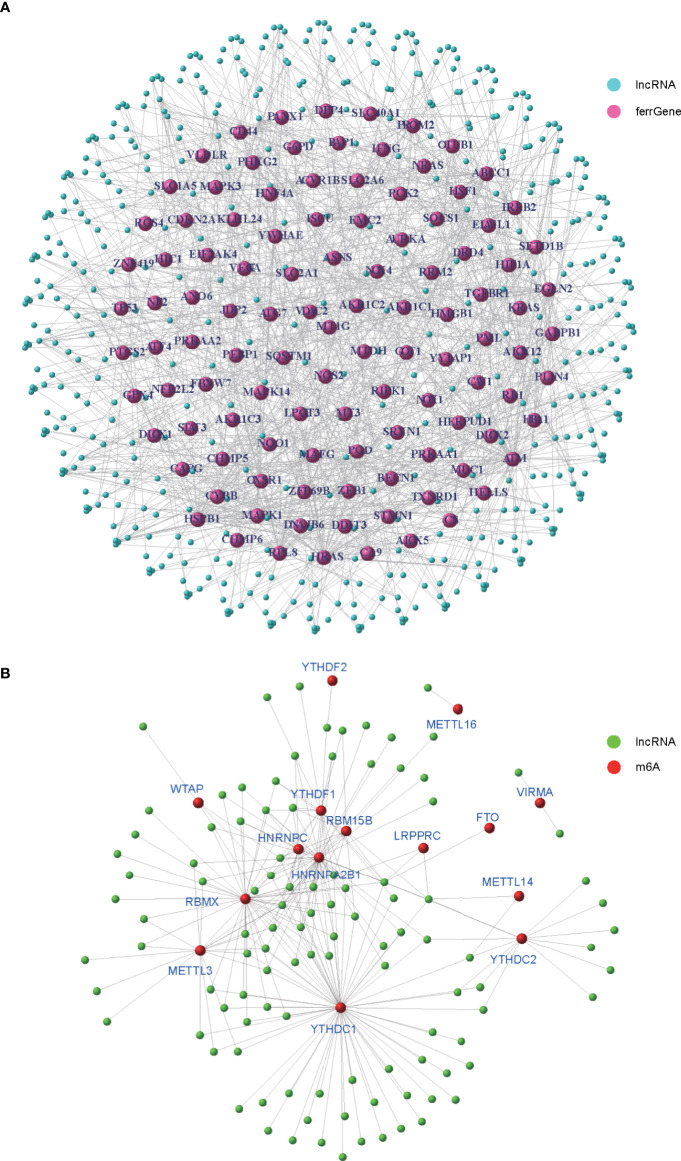
Generation of mfrlncRNA. **(A)** The coexpression network of 201 ferroptosis-related genes (ferrGenes) and 14,080 lncRNAs, named as ferroptosis-related lncRNA (frlncRNA, *R* = 0.5, *p* = 0.001). **(B)** The coexpression network of 23 m6A regulators and 502 frlncRNAs, named as m6A- and ferroptosis-related lncRNA (mfrlncRNA, *R* = 0.5, *p* = 0.001).

To identify m6A- and ferroptosis-related lncRNAs (mfrlncRNA), correlation coefficients between 14,080 lncRNAs and 201 ferroptosis-related genes (ferrGene) were compared according to coexpression, and 502 ferroptosis-related lncRNAs (frlncRNAs) were identified (**Figure 1A**, *R* = 0.5, *p* = 0.001). Using the same method, 502 frlncRNAs and 23 m6A regulators were further coexpressed to obtain mfrlncRNAs (**Figure 1B**, *R* = 0.5, *p* = 0.001), and a total of 108 mfrlncRNAs were identified.

**Figure 2:**
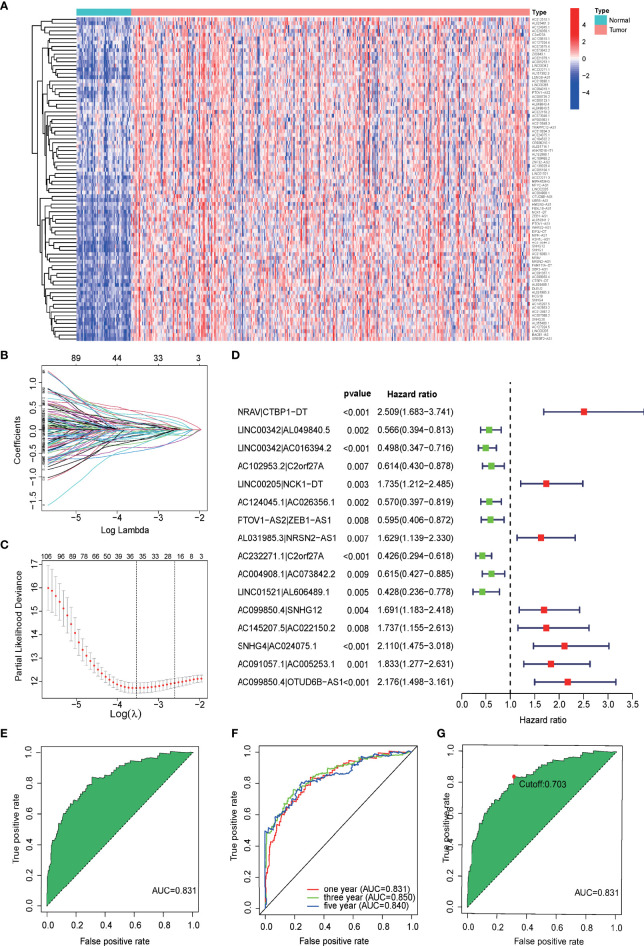
Construction of a prognostic signature based on mfrlncRNA pairs. **(A)** Heatmap of 84 differentially expressed mfrlncRNAs between normal and tumor groups (FDR = 0.05, logFC = 1). **(B)** Crossvalidation for tuning parameter selection in the LASSO model. **(C)** LASSO coefficient profiles of 35 mfrlncRNA pairs. **(D)** The forest map indicated 16 mfrlncRNA pairs identified by Cox regression, used for the construction of the mfrlncRNA signature. **(E)** The ROC of mfrlncRNA signature, whose maximum AUC value was 0.831. **(F)** The 1-, 3-, and 5-year ROC of the optimal model was 0.831, 0.850, and 0.840, respectively. **(G)** Risk scores for 365 patients with HCC; the maximum inflection point was the cutoff point (cutoff = 0.703). mfrlncRNA, m6A- and ferroptosis-related lncRNA. LASSO, least absolute shrinkage and selection operator; OS, overall survival; HCC, hepatocellular carcinoma; ROC, receiver operating characteristic curve; AUC, area under the curve.

According to the difference analysis, 84 differentially expressed mfrlncRNAs were identified between the normal and tumor groups (**Figure 2A**, FDR = 0.05, logFC = 1). After the construction of 1,619 mfrlncRNA pairs, a univariate Cox analysis was performed to screen 129 prognostic mfrlncRNA pairs (*p* = 0.01). Subsequently, 35 more precise mfrlncRNA pairs were identified according to LASSO analysis (**Figures 2B, C**). The mfrlncRNA signature was constructed based on 16 mfrlncRNA pairs using Cox proportional hazard regression (**Figure 2D**).

The expression of each mfrlncRNA pair multiplied by the coefficient was calculated to obtain risk scores. A ROC was drawn, and an AUC was calculated to demonstrate the good accuracy of the mfrlncRNA signature (**Figure 2E**). The 1-, 3-, and 5-year ROC curve of the optimal model was 0.831, 0.850, and 0.840, respectively, proving that the diagnostic value of the mfrlncRNA signature was superior (**Figure 2F**). Maximization of the sum of sensitivity and specificity was taken as the optimal cutoff point (**Figure 2G**, cutoff = 0.703). Based on the cutoff point, 365 samples were stratified into 146 high- and 219 low-risk samples.

### Prognostic value of mfrlncRNA signature

**Figure 3:**
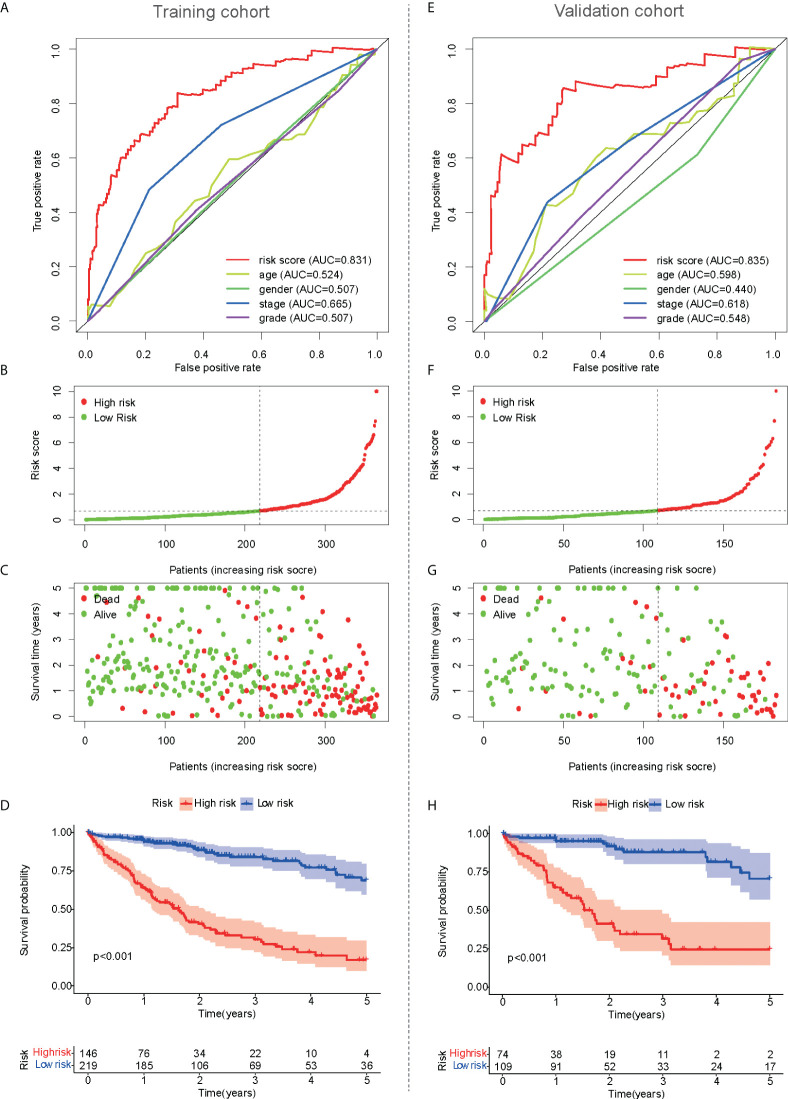
Prognostic value of mfrlncRNA signature. **(A)** A comparison of 1-year ROC curves with other common clinical traits indicated the advantage of the risk score in the training cohort. **(B, C)** Risk scores **(B)** and survival outcome **(C)** of each sample in the training cohort were shown. **(D)** Kaplan–Meier analysis for OS of HCC patients based on the risk stratification in the training cohort. **(E–H)** Prognostic value of mfrlncRNA signature in the validation cohort. HCC, hepatocellular carcinoma; OS, overall survival.

The prognostic value of the risk score was analyzed in the training cohort. Based on the clinical data of 365 HCC patients, the independent prognostic analysis showed that the area under the ROC curve of the risk score was significantly better than that of other clinical traits (**Figure 3A**). Therefore, the risk score calculated from the mfrlncRNA signature had a better prognostic value. The risk assessment, risk score, and survival for each case are shown in **Figures 3B, C**, respectively. This showed that as the patient’s risk score increased, the patient’s survival time decreased and the number of deaths increased, proving that a higher risk score indicated poor survival. OS of HCC patients was compared according to Kaplan–Meier analysis, and patients with a low-risk score had a better prognosis than those with a high-risk score (**Figure 3D**). Similar results were observed in the validation cohort (**Figures 3E–H**).

**Figure 4:**
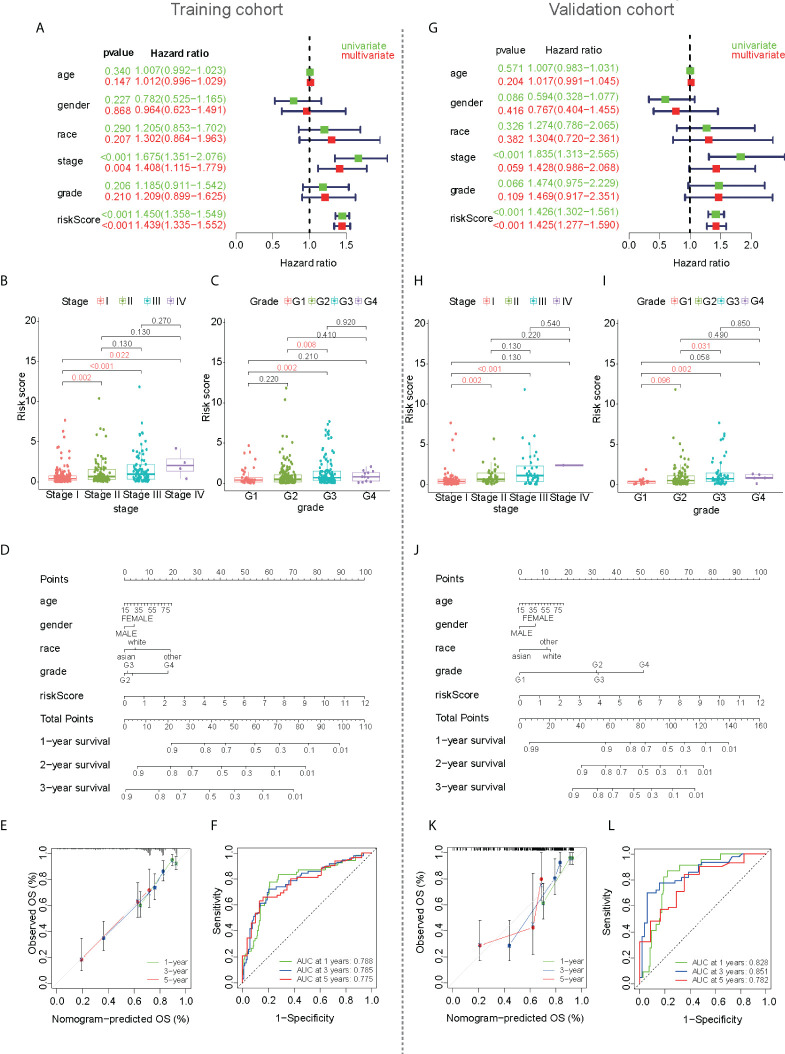
Development of a nomogram based on mfrlncRNA signature used to predict the survival of HCC patients. **(A)** Cox regression for the mfrlncRNA signature in the training cohort. Red represents multivariate Cox regression and green represents univariate Cox hazard ratio analysis. **(B, C)** The differences in stage **(B)** and tumor grade **(C)** between high- and low-risk populations in the training cohort. **(D)** Nomogram for predicting the 1-, 3-, and 5-year overall survival of patients with HCC in the training cohort. **(E)** Calibration curve for predicting OS in the training cohort. **(F)** The 1-, 3-, and 5-year ROC of the nomogram model were 0.788, 0.785, and 0.775 in the training cohort, respectively. **(G–L)** The development of a nomogram in the validation cohort. HCC, hepatocellular carcinoma; ROC, receiver operating characteristic curve.

Correlations between clinical traits and prognostic values were also analyzed in the training cohort. The forest plot showed that clinical staging and risk score were independent prognostic factors, and poor prognosis was significantly associated with high-risk scores in both the univariate and multivariate Cox regression analyses (**Figure 4A**). The scatter plot showed that there were differences in the risk scores of different TNM stages, indicating a correlation between the risk scores and TNM stage (**Figure 4B**). Therefore, TNM staging was not included in the subsequent nomogram model. The tumor grade was similar to the TNM stage, and the risk score also correlated with the tumor grade (**Figure 4C**). To predict the patients’ 1-, 3-, and 5-year survival rates, a nomogram plot was drawn based on clinical traits (**Figure 4D**). The total score was determined based on individual scores and contributed to the survival prediction. The calibration curves of the model showed a high degree of agreement between the predicted and observed survival probabilities in the training cohort (**Figure 4E**). The AUC values (**Figure 4F**) were all greater than 0.8, indicating that the nomogram based on the mfrlncRNA signature had good accuracy. Although the TNM stage had little significance for survival prediction according to multivariate Cox regression, the mfrlncRNA signature still showed good prognostic value in the validation cohort (**Figures 4G–L**).

### Immune infiltration and efficacy prediction of mfrlncRNA signature

**Figure 5:**
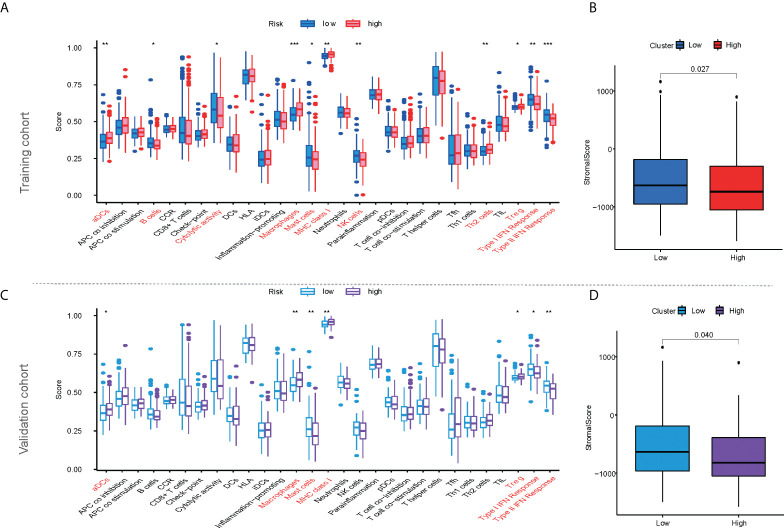
The characteristics of immune infiltration based on mfrlncRNA signature. **(A)** Differential analysis of 29 immune signatures according to ssGSEA analysis between high- and low-risk populations in the training cohort. **(B)** Differential analysis of stromal scores according to ESTIMATE analysis between high- and low-risk populations in the training cohort. **(C, D)** The characteristics of immune infiltration based on mfrlncRNA signature in the validation cohort. The asterisks represented the statistical *p*-value (*p* > 0.05; ^*^ *p* < 0.05; ^**^ *p* < 0.01; ^***^ *p* < 0.001). ssGSEA, single-sample gene-set enrichment analysis; ESTIMATE, Estimation of STromal and Immune cells in MAlignant Tumour tissues using Expression data.

According to ssGSEA analysis, 11 of the 29 immune signatures showed significant differences in immune infiltration between the high- and low-risk populations in the training cohort (**Figure 5A**). In addition, the stromal score was higher in the low-risk population than in the high-risk population according to the ESTIMATE analysis (**Figure 5B**, *p* < 0.05). The same calculation was applied to the validation and ICGC cohorts, and the results were similar to those of the training cohort. Four immune signatures showed significant dissimilarities in the training, validation, and ICGC cohorts, such as activated dendritic cells (aDCs), macrophages, major histocompatibility complex (MHC) class I, and regulatory T cells (Tregs) (**Figure 5C**; **Supplementary Figure S1A**). Similar to the training cohort, the stromal score in the validation cohort was higher in the low-risk population (**Figure 5D**).

**Figure 6:**
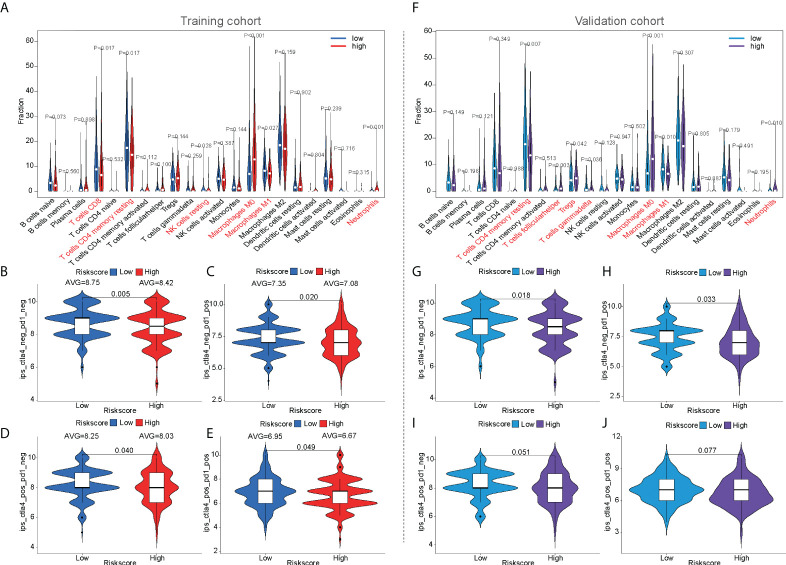
The prediction of immunotherapeutic response based on mfrlncRNA signature. **(A)** The infiltration of 22 immune cells according to CIBERSORT analysis between high- and low-risk populations in the training cohort. **(B–E)** IPS between high- and low-risk populations in the training cohort. **(F)** The infiltration of 22 immune cells in the validation cohort. **(G–J)** IPS between high- and low-risk populations in the validation cohort. CIBERSORT, Cell-type Identification by Estimating Relative Subsets of RNA Transcripts; IPS, immunophenoscore.

The CIBERSORT algorithm was used to calculate the infiltration of 22 immune cells to compare immune infiltration between the high- and low-risk populations in the training cohort (**Figure 6A**). Six immune cells were confirmed to have different infiltrations between high- and low-risk populations in both the training and validation cohorts, such as CD8^+^ T cells, memory resting CD4^+^ T cells, resting NK cells, M0 macrophages, M1 macrophages, and neutrophils. Similar results were observed in the validation cohort (**Figure 6F**). Four immune cells were confirmed to have different infiltrations between high- and low-risk populations in the training and ICGC cohorts, such as memory B cells, naive CD4^+^ T cells, activated NK cells, and activated dendritic cells (**Supplementary Figure S1B**).

For the prediction of immunotherapeutic response based on the mfrlncRNA signature, the immunophenoscore (IPS) of patients who received different treatments was calculated, such as patients with no treatment, anti-CTLA4 monotherapy, anti-PD1 monotherapy, or combination therapy. In the training cohort, the violin diagram showed that immune efficacy was dissimilar between the high- and low-risk populations (**Figures 6B–E**). Moreover, patients in the low-risk population had higher scores, indicating that low-risk patients had greater efficacy in receiving immunotherapy and were more suitable for immunotherapy. Unfortunately, in the validation cohort, only the immune response of patients without treatment or with anti-CTLA4 monotherapy was similar to that in the training cohort. (**Figure 6G–J**). In addition, we also analyzed the guiding value of the mfrlncRNA signature for tumor immune dysfunction and exclusion (TIDE), another immune response indicator, suggesting that the patients with high risk had more potential for immune escape and worse efficacy in receiving immunotherapy (**Supplementary Figure S1C**). Furthermore, similar results were obtained in the ICGC cohort (**Supplementary Figure S1D**), which was consistent with previous predictions of immune efficacy based on IPS.

**Figure 7:**
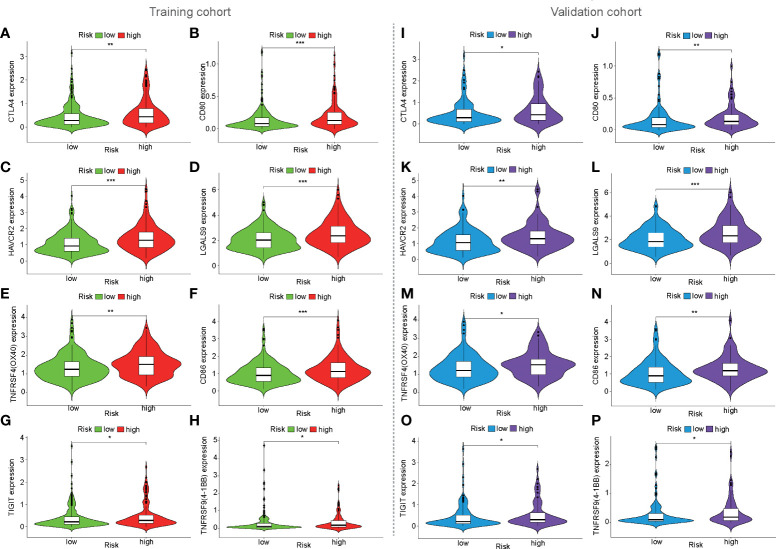
Expression of immune checkpoints based on mfrlncRNA signature. **(A–H)** The expression of immune checkpoints between high- and low-risk populations in the training cohort. **(I–P)** Expression of immune checkpoints in the validation cohort. (*p* > 0.05; *p < 0.05; **p < 0.01; ***p < 0.001).

To preliminarily analyze the reasons for the difference in immune efficacy between the high- and low-risk populations, the expression of immune checkpoints was analyzed in three cohorts. It was found that eight immune checkpoints were upregulated in the high-risk population in the training, validation, and ICGC cohorts, such as CTLA4, CD80, HAVCR2 (TIM3), LGALS9, CD86, TNFRSF4 (OX40), TNFRSF9 (4-1BB), and TIGIT (**Figures 7**, **S2A–H**). Furthermore, VSIR expression (*p* < 0.05, **Figure 7H**) was also upregulated in the high-risk population in the ICGC cohort (**Supplementary Figure S2I**).

**Figure 8:**
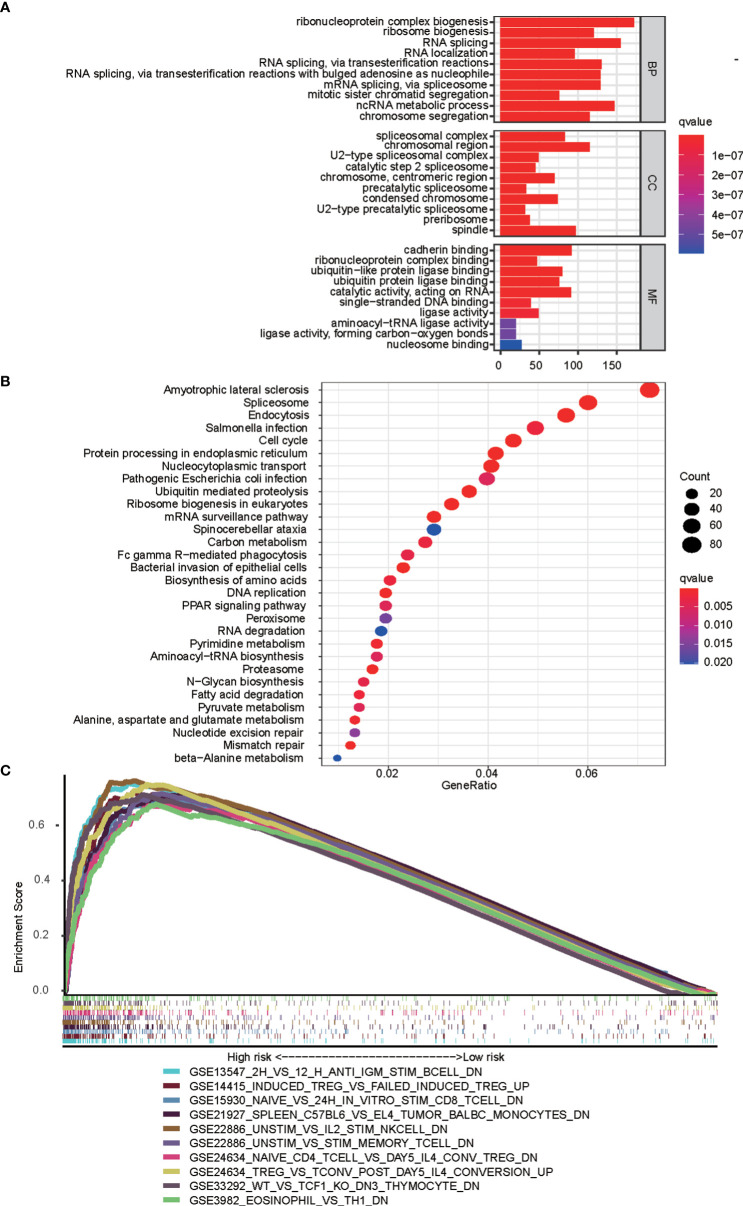
Functional analyses based on mfrlncRNA signature. **(A)** Significantly enriched GO terms according to differential genes between high- and low-risk populations. **(B)** Top 30 most enriched KEGG pathways of the common differently genes between high- and low-risk populations **(C)** GSEA enrichment analysis showing the activation states of biological pathways in high- and low-risk populations. GO, gene ontology; KEGG, Kyoko Encyclopaedia of Genes and Genomes; GSEA, Gene Set Enrichment Analysis.

GO and KEGG analyses were performed to reveal immune function differences between high- and low-risk subgroups. GO enrichment analysis indicated that the top 10 enrichment results in the three categories of biological process, cellular component, and molecular function, such as ribonucleoprotein complex biogenesis, spliceosomal complex, and cadherin binding (**Figure 8A**). According to the KEGG enrichment analysis, the bubble map shows the top 30 enrichment pathways, including spliceosome, nucleocytoplasmic transport, and cell cycle (**Figure 8B**). GSEA was used to verify the immune signatures of the high-risk population, and the first 10 active pathways in the high-risk population were visualized to form a GSEA enrichment map, indicating that the high-risk population was significantly enriched with these genes. Briefly, the results of functional enrichment analysis revealed potential pathways or mechanisms that were activated during tumorigenesis and development, which may help us to evaluate the prognosis of HCC patients (**Figure 8C**).

### Chemotherapy response prediction based on mfrlncRNA signature

**Figure 9:**
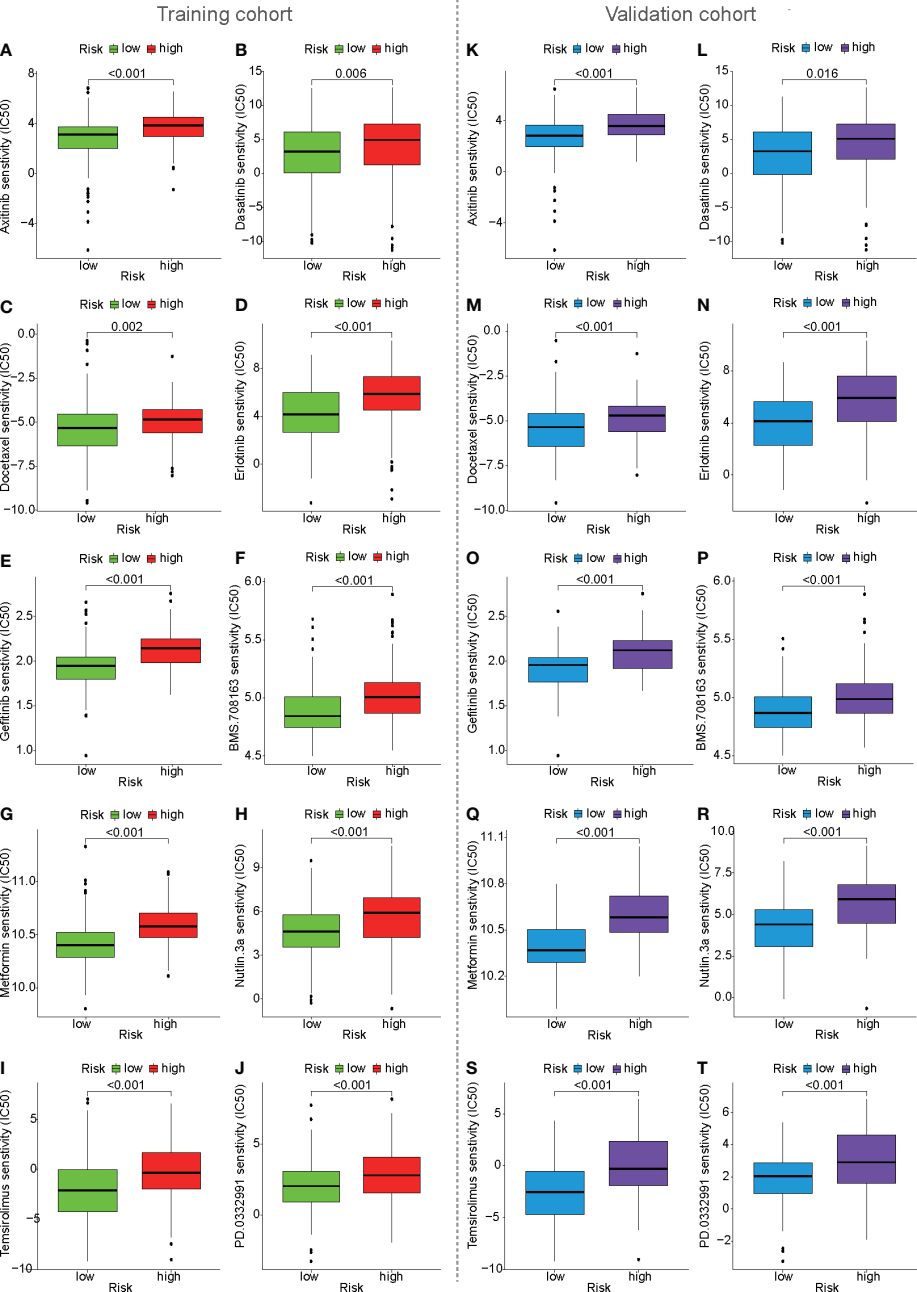
Prediction of drug sensitivity based on mfrlncRNA signature. **(A–J)** Prediction of drug sensitivity between high- and low-risk populations in the training cohort. **(K–T)** Prediction of drug sensitivity in the validation cohort.

To predict the chemotherapy response in the training cohort, antitumor drugs for liver cancer were selected for chemotherapy response prediction. IC_50_ was used to represent drug sensitivity. The IC_50_ of some HCC drugs, such as axitinib, dasatinib, docetaxel, erlotinib, gefitinib, BMS.708163, metformin, nutlin.3a, PD.0332991, and temsirolimus, were found to be higher in the high-risk population than in the low-risk population, suggesting that high-risk patients have poorer efficacy (**Figures 9A–J**). The same results were also observed in the validation cohort (**Figures 9K–T**). In the ICGC cohort, it is surprising that the IC_50_ of some HCC drugs, such as BMS.708163 and gefitinib, were found to be higher in the high-risk population than in the low-risk population (**Supplementary Figures S2J–M**), which was consistent with previous predictions of chemotherapy response in the training and validation cohorts.

## Discussion

**Figure 10:**
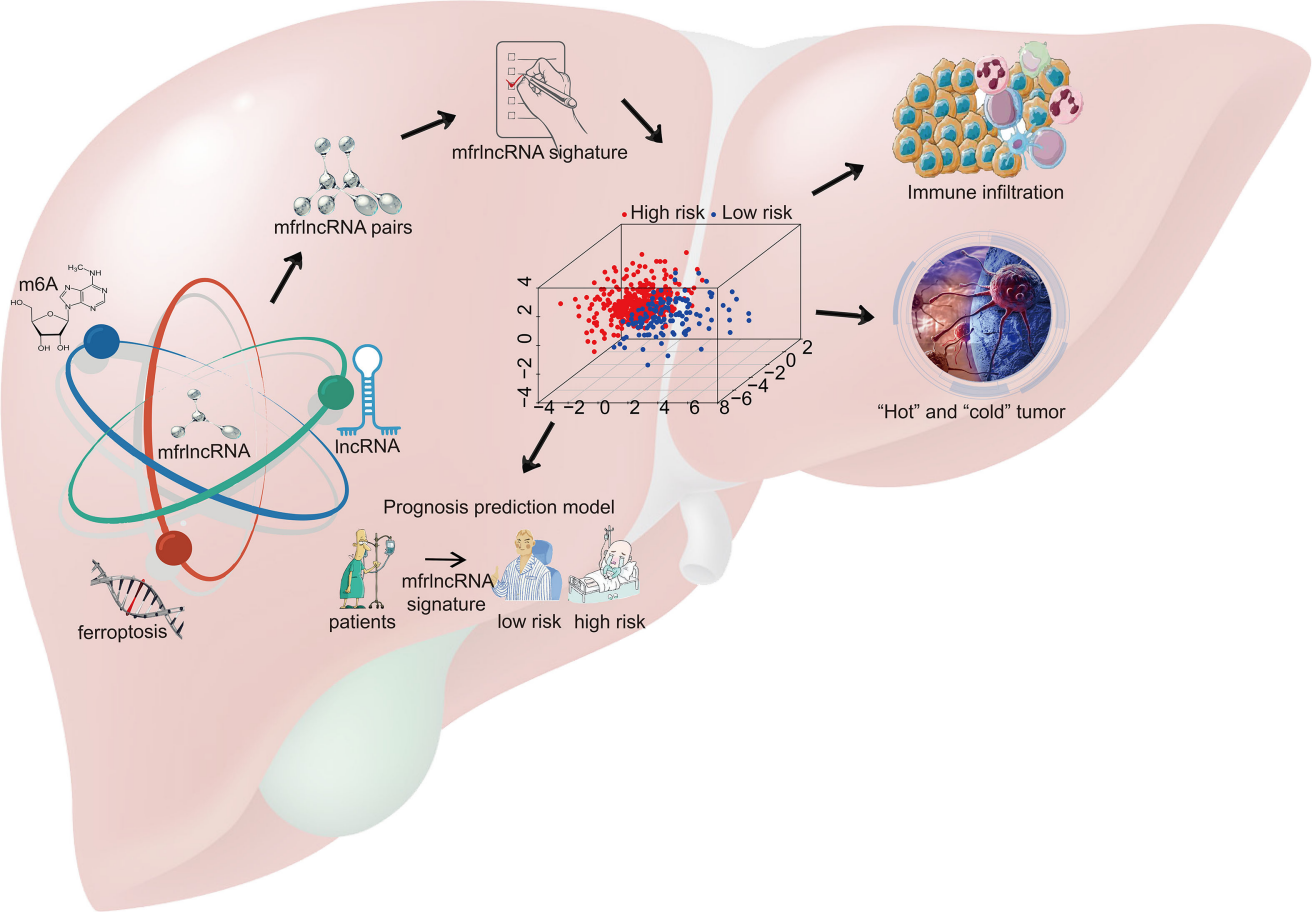
Schematic diagram. A group of specific lncRNAs associated with both m6A regulators and ferroptosis-related genes was identified using correlation analysis and named m6A- and ferroptosis-related lncRNA (mfrlncRNA). The mfrlncRNA signature was constructed based on mfrlncRNA pairs and patients were stratified into high- and low-risk populations. Through further exploration, the prognostic value of the mfrlncRNA signature was evaluated as well as immune infiltration, immune efficacy, and drug sensitivity.

In this study, we first identified a group of specific lncRNAs associated with both m6A regulators and ferroptosis-related genes and then constructed prognosis-related mfrlncRNA pairs. Based on this, the mfrlncRNA signature was constructed using the LASSO analysis and Cox regression. Notably, the risk score of patients was proven to be an independent prognostic factor and was better than the TNM stage and tumor grade. Moreover, patients with high-risk scores had lower survival rates, higher infiltration of immunosuppressive cells (macrophages and Tregs), lower infiltration of cytotoxic immune cells (NK cells), poorer immune efficacy, higher IC_50_, and enrichment of the induced Treg pathway. In conclusion, the mfrlncRNA signature was shown to contribute to the survival prediction and risk stratification of patients with HCC (**Figure 10**).

As a research hotspot in the field of cancer, diagnosis, and treatment (35), lncRNAs are associated with tumorigenesis and metastasis through aberrant expression and mutations (36). Some lncRNAs act as tumor suppressors, whereas others promote tumor development (37). Some lncRNAs have been reported to be potential biomarkers for patients with HCC alone, either with high sensitivity or in combination with other molecules to improve specificity (38). Studies have shown that lncRNA-D16366 is a potential biomarker for the diagnosis and prognosis of HCC (39) and that a 25-lncRNA prognostic signature can be identified for early recurrence in HCC (40). Although the lncRNA signature showed some prognostic benefits for patients with HCC, the large number of variables and lack of precision were its drawbacks. Inspired by the construction of a prognostic model based on the combination of two prognostic biomarkers (41), we attempted to combine m6A and ferroptosis, which have been reported to be prognostic biomarkers (42, 43), to improve the accuracy of the lncRNA prognostic signature. In this study, a group of specific lncRNAs associated with both m6A regulators and ferroptosis-related genes was identified (**Figures 1**, **2A**) and used to construct the mfrlncRNA signature. Based on the combination of three prognostic biomarkers, the mfrlncRNA signature had better accuracy, with AUCs for 1-, 3-, and 5-year survival rates of 0.831, 0.850, and 0.840, respectively (**Figures 2E–G**). In addition, the specific expression of identified lncRNAs is required for the construction of the common lncRNA signature. As a result, normalization needs to be performed to decrease batch effects before clinical application (44). However, this problem can be solved by using lncRNA pairs, as Hong et al. constructed immune-related lncRNA pairs to perform prognostic analysis for HCC (45). This method was also applied in this study. The mfrlncRNA signature was constructed using mfrlncRNA pairs, which needed to be compared to the pairs instead of an exact expression of every lncRNA (**Figures 2B–D**). In conclusion, it was a great innovation to construct an mfrlncRNA signature, which had greater predictive power than common clinical prognostic models.

Recently, a variety of clinical traits, such as age, sex, tumor grade, TNM stage, and treatment, have been used to construct a nomogram to predict the survival of patients with HCC. For example, nomogram development was constructed using clinical traits such as treatment, survival, and prognostic factors of HCC (46). A practical nomogram was constructed to predict the prognosis of young patients with HCC after curative liver resection (47). Therefore, it is feasible to evaluate the prognosis of patients with HCC based on clinical traits. Compared to the above studies, our prognostic model is more valuable because the risk score calculated based on the mfrlncRNA signature as an independent prognostic factor was better than common clinical traits such as TNM stage and tumor grade in predicting the OS for HCC (**Figures 3A, E**). The mfrlncRNA signature contributed to building a nomogram with better accuracy in predicting the survival rate of patients with HCC (**Figure 4**). In addition, the mfrlncRNA signature has great advantages because it contributes to the risk stratification of patients with HCC (**Figures 3B, C, F, G**). Tumor stratification is of great importance for patients to achieve better clinical outcomes (48). For example, integrative molecular HCC subtypes based on significantly mutated genes were reported to provide potential directions for future therapeutic efforts (49). HCC subtypes based on immunologic genes contribute to the selection of HCC treatment modalities (50). In this study, we identified two distinct risk populations with different prognoses, which may help develop different therapeutic strategies (**Figures 3D, H**). For example, risk stratification based on the mfrlncRNA signature has been confirmed to contribute to the prediction of chemotherapy response. Based on drug sensitivity analysis, we explored commonly used chemotherapeutic agents for HCC, such as axitinib, dasatinib, docetaxel, erlotinib, gefitinib, metformin, nutlin.3a, and temsirolimus (51–58). The results indicated that patients with high-risk scores had a lower sensitivity to multiple chemotherapy drugs for HCC (**Figure 9**). Briefly, based on the mfrlncRNA signature, we can allocate patients to more reasonable curative procedures that provide a survival benefit, thus improving the survival rate of patients with HCC.

HCC is a representative inflammation-induced cancer, and immune cells have been reported to play pro- or antitumor roles in HCC (59). For example, NK cells have strong antitumor activity and contribute to immunotherapeutic approaches for HCC treatment (60). It has been reported that macrophages, which are proinflammatory, promote tumor formation by suppressing the antitumor immune response (61). The infiltration of Treg cells has also been reported to promote tumor formation (62). As critical regulators of gene expression in the immune system (63), lncRNAs are of great importance in directing the development of diverse immune cells and in controlling dynamic transcriptional programs (64). For example, lncRNA MIAT correlates with immune infiltrates and drug reactions in HCC (65). lncRNA TCL6, which was shown to correlate with immune cells, showed a poorer prognosis in patients with breast cancer (66). In this study, the mfrlncRNA signature, which was constructed using a group of specific lncRNAs, was able to evaluate the immune infiltration of patients with HCC. Based on the risk stratification, it was exciting that patients with high-risk scores had higher infiltration of immunosuppressive cells, such as macrophages and Tregs, as well as lower infiltration of cytotoxic immune cells, such as NK cells (**Figures 4**, **5A, F**). Therefore, patients with a high-risk score appeared to have an inflammatory microenvironment that contributed to the development of HCC. In addition, lncRNAs may contribute to the discrimination of “cold” and “hot” HCC tumors. For example, it was proved that necroptosis-related lncRNAs can be used to distinguish the cold and hot tumors in gastric cancer (67). As opposed to a hot tumor, the cold tumors lacked T-cell infiltration and were involved in initial resistance to immune checkpoint inhibitors (68). In this study, we found that CD8^+^ T cells, memory resting CD4^+^ T cells, and resting NK cells were downregulated in patients with a high-risk score than in those with a low-risk score. This suggested that high- and low-risk populations based on mfrlncRNA signature correspond to cold and hot tumors, respectively. Patients with high-risk scores had poorer immune efficacy than those in the low-risk population (**Figures 6B–E**, **G–J**). Furthermore, the expression of immune checkpoints was analyzed in this study to preliminarily explore the reasons for the difference in immune efficacy between the high- and low-risk populations. In recent years, inhibitory immune checkpoints, such as CTLA-4, have been shown to suppress antitumor immune responses in HCC (69). Recent studies have shown that immune checkpoint inhibitors have made an indelible mark in the field of cancer immunotherapy (70), and tumor immunotherapy has proven to be of great importance (71). Therefore, it is important to monitor the expression levels of immune checkpoints to evaluate immunotherapy efficacy. In our study, we found that patients with high-risk scores had an upregulation of immune checkpoints (**Figure 6**), which means that the mfrlncRNA signature has a potential predictive significance for the efficacy of immunotherapy. Briefly, the results showed that the mfrlncRNA signature could predict survival rates and efficacy in patients with HCC.

External validation is necessary to determine the reproducibility and generalizability of the prediction model for HCC patients (72). Therefore, in addition to the validation cohort, external validation, which makes our conclusions more convincing, was performed based on HCC data from the ICGC database. However, in the available HCC data of the ICGC database, two lncRNAs, AC026356.1 and SNHG4 in the mfrlncRNA signature, were not detected. Corresponding mRNA precursors or small nuclear RNAs with the same transcription efficiency were used to replace them respectively, so as to construct the risk model successfully. AC026356.1 (ENSG00000274964), as a novel transcript, is a sense intronic to BICD1. Herein, AC026356.1 was replaced by BICD1. SNORA74A was encoded by the second intron of host gene SNHG4 (also named U19H). Therefore, SNORA74A was used to replace SNHG4 (30, 31). The results of the external validation nicely duplicated and confirmed the previous immune infiltration, immune efficacy, and chemotherapy responses in the training and validation cohorts, which suggested that the mfrlncRNA signature was able to excellently predict the efficacy of patients with HCC.

Admittedly, the present study has several limitations. First, we constructed the validation cohort by randomly sampling half of the HCC samples from TCGA database, and then used HCC samples from the ICGC database as external validation (ICGC cohort). However, further experimental studies are required to verify the reliability of these results. In addition to this, more sufficient samples need to be collected to confirm the value of the mfrlncRNA signature in the future. Second, the mfrlncRNA signature has a good prognostic value for diagnosis and prognosis, but the value of early diagnosis needs to be further studied. Third, no suitable data from the GEO database are available to validate our results. Fourthly, since this study is purely a biological information analysis, more cohort studies are required to confirm the outcomes of the clinical model before it is applied to the clinic. Finally, there are some differences between the results of the training cohort, the validation cohort, and even the ICGC cohort. The potential reason behind this phenomenon probably lies in the small sample sizes of the verification cohort and the ICGC cohort. After all, the sample size of the validation cohort was only half of the training set, at 183 cases. The sample size of the ICGC cohort was only 161 cases. However, the verification results were basically in a reasonable and acceptable range.

In conclusion, the mfrlncRNA signature based on 16 optimal mfrlncRNA pairs not only has good prognostic value and prediction accuracy but also helps in risk stratification and predicts the immune efficacy and drug sensitivity of patients with HCC. As a result, the mfrlncRNA signature provides new clues for identifying cold and hot tumors to optimize the status of immune surveillance and might have crucial implications for individualized therapy to improve the survival rate of patients with HCC.

## Data availability statement

The datasets used in the current study and related scripts of bioinformatics analysis are available from the corresponding author upon reasonable request.

## Ethics statement

The studies involving human participants were reviewed and approved by The Clinical Research Ethics Committee of the Affiliated Hospital of Nantong University. Written informed consent for participation was not required for this study in accordance with the national legislation and the institutional requirements. Written informed consent was not obtained from the individual(s) for the publication of any potentially identifiable images or data included in this article.

## Author contributions

HX and HZ have access to all the data in this study and take responsibility for the integrity and accuracy of the data analyses. HZ, MY, MS, and YL: study concept and design. HX, LS, and ZD: drafted the manuscript. HX, CC, XC, YW, and HJ: statistical analysis. HZ, DY, and PW: supervised the study. All authors have read and approved the final manuscript.

## Funding

This work was supported by the National Natural Science Foundation of China (81401988), China Postdoctoral Science Foundation (2019M661907), Jiangsu Postdoctoral Science Foundation (2019K159, 2019Z153), and General Project of Jiangsu Provincial Health Committee (H2019101).

## Acknowledgments

The authors acknowledge The Cancer Genome Atlas (TCGA) database for the convenience of this research.

## Conflict of interest

The authors declare that the research was conducted in the absence of any commercial or financial relationships that could be construed as a potential conflict of interest.

## Publisher’s note

All claims expressed in this article are solely those of the authors and do not necessarily represent those of their affiliated organizations, or those of the publisher, the editors and the reviewers. Any product that may be evaluated in this article, or claim that may be made by its manufacturer, is not guaranteed or endorsed by the publisher.
