# Supplementary material for: Identification of m6A- and ferroptosis-related lncRNA signature for predicting immune efficacy in hepatocellular carcinoma
Source: Front Immunol. 2022 Aug 11;13:914977. doi: 10.3389/fimmu.2022.914977 (PMC9402990; doi:10.3389/fimmu.2022.914977)
Supplement: Supplementary file 1 [file DataSheet_1.zip › Supplementary Material/Figure S1.pdf]

Figure S1

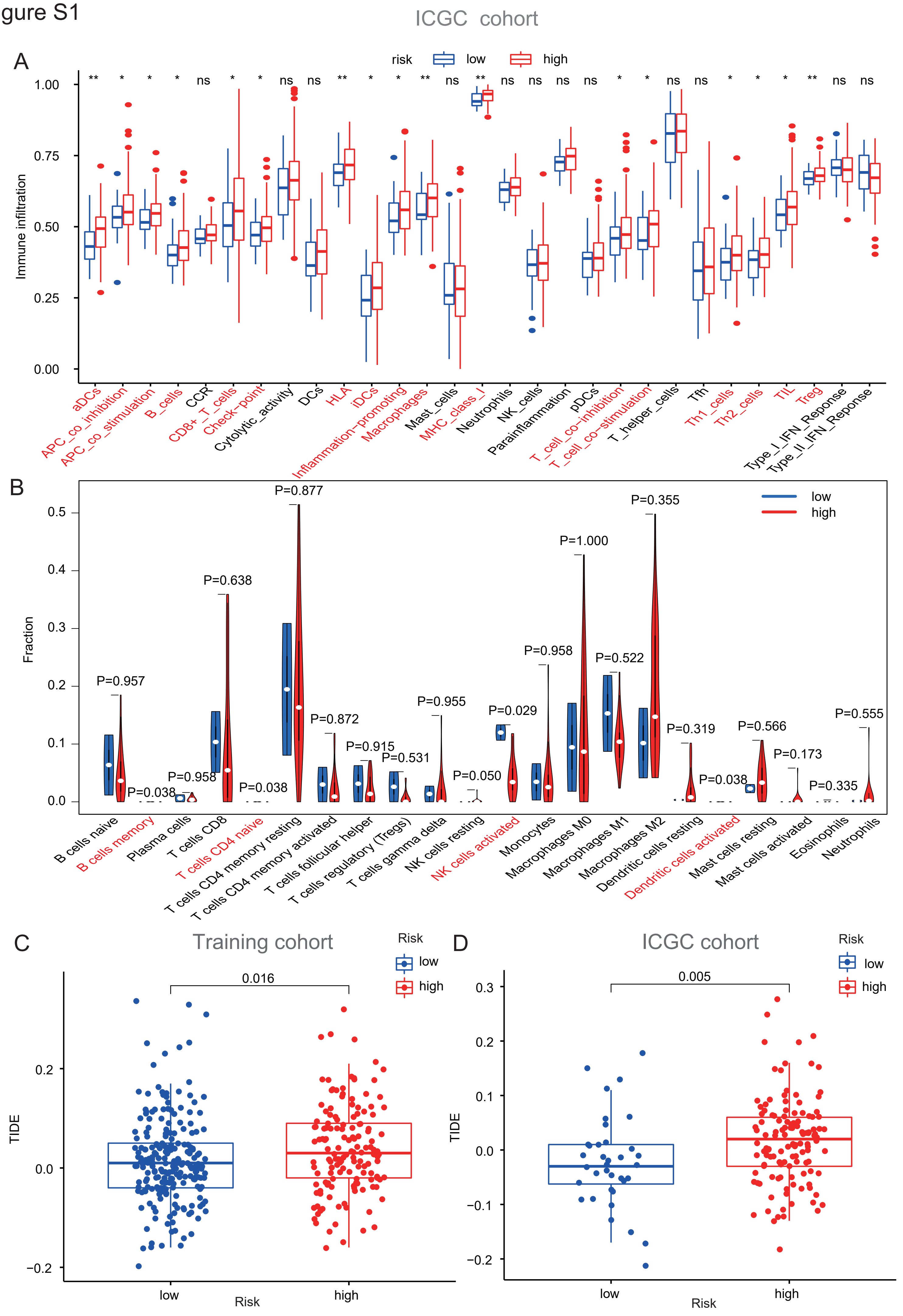

Figure S1. Analysis of immune infiltration and immune efficacy in ICGC cohort.

A. Differential analysis of 29 immune signatures according to ssGSEA analysis between high- and low-risk populations in ICGC cohort. B. The infiltration of 22 immune cells according to CIBERSORT analysis between high- and low-risk populations in ICGC cohort. C-D. Differential analysis of TIDE score between high- and low-risk populations in training cohort and ICGE cohort, respectively. The asterisks represented the statistical p value (*, P < 0.05; **,P < 0.01; ***, P < 0.001). ssGSEA, single-sample gene-set enrichment analysis. CIBERSORT, Cell-type Identification by Estimating Relative Subsets of RNA Transcripts. TIDE, Tumor Immune Dysfunction and Exclusion.
