# Supplementary material for: Identification of m6A- and ferroptosis-related lncRNA signature for predicting immune efficacy in hepatocellular carcinoma
Source: Front Immunol. 2022 Aug 11;13:914977. doi: 10.3389/fimmu.2022.914977 (PMC9402990; doi:10.3389/fimmu.2022.914977)
Supplement: Supplementary file 1 [file DataSheet_1.zip › Supplementary Material/Figure S2.pdf]

Figure S2

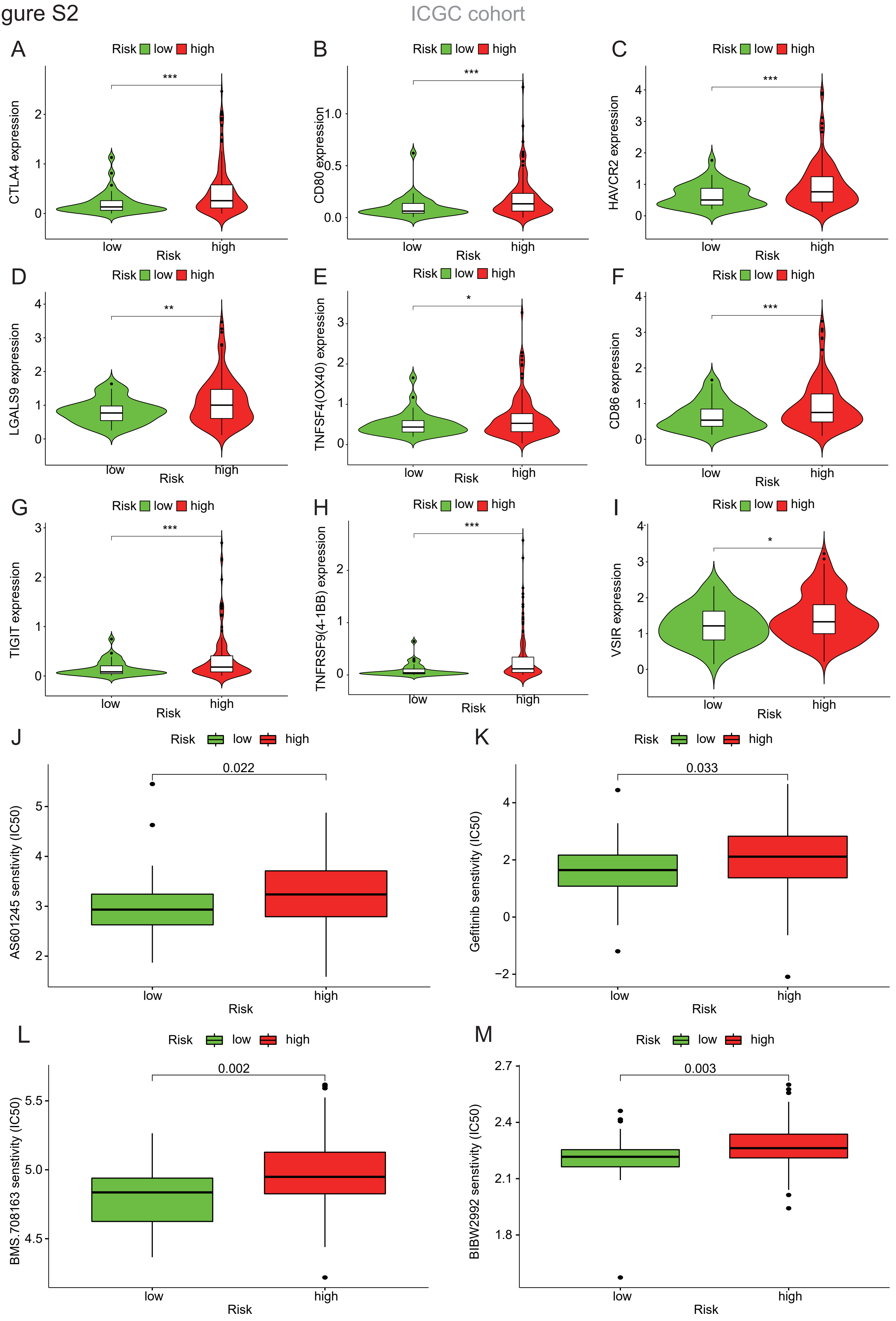

Figure S2. Analysis of immune checkpoints and drug sensitivity in ICGC cohort.

A-H. The expression of immune checkpoints between high- and low-risk populations in ICGC cohort. I-Q. Prediction of drug sensitivity between high- and low-risk populations in ICGC cohort. The asterisks represented the statistical p value (*, P < 0.05; **,P < 0.01; ***, P < 0.001).
