# Supplementary material for: Identification of m6A- and ferroptosis-related lncRNA signature for predicting immune efficacy in hepatocellular carcinoma
Source: Front Immunol. 2022 Aug 11;13:914977. doi: 10.3389/fimmu.2022.914977 (PMC9402990; doi:10.3389/fimmu.2022.914977)
Supplement: Supplementary file 1 [file DataSheet_1.zip › Supplementary Material/Table S1.DOCX]

| **Characteristic** | **Training Cohort (n=365)** | **Validation Cohort (n=183)** | **Total (n=548)** | **P-value** |
| --- | --- | --- | --- | --- |
| Age |  |  |  |  |
| <65 | 216 (59.2%) | 101 (55.2%) | 317 (57.8%) |  |
| >=65 | 149 (40.8%) | 82 (44.8%) | 231 (42.2%) | 0.424 |
| Gender |  |  |  |  |
| Female | 119 (32.6%) | 54 (29.5%) | 173 (31.6%) |  |
| Male | 246 (67.4%) | 129 (70.5%) | 375 (68.4%) | 0.524 |
| Race |  |  |  |  |
| White | 182 (49.9%) | 92 (50.3%) | 274 (50%) |  |
| Asian | 155 (42.5%) | 74 (40.4%) | 229 (41.8%) |  |
| Other | 18 (4.9%) | 13 (7.1%) | 31 (5.7%) |  |
| Unknow | 10 (2.7%) | 4 (2.2%) | 14 (2.6%) | 0.744 |
| Stage |  |  |  |  |
| Stage I | 170 (46.6%) | 83 (45.4%) | 253 (46.2%) |  |
| Stage II | 84 (23%) | 45 (24.6%) | 129 (23.5%) |  |
| Stage III | 83 (22.7%) | 41 (22.4%) | 124 (22.6%) |  |
| Stage IV | 4 (1.1%) | 1 (0.5%) | 5 (0.9%) |  |
| Unknow | 24 (6.6%) | 13 (7.1%) | 37 (6.8%) | 0.960 |
| Grade |  |  |  |  |
| G1 | 55 (15.1%) | 22 (12%) | 77 (14.1%) |  |
| G2 | 175 (47.9%) | 100 (54.6%) | 275 (50.2%) |  |
| G3 | 118 (32.3%) | 52 (28.4%) | 170 (31%) |  |
| G4 | 12 (3.3%) | 5 (2.7%) | 17 (3.1%) |  |
| Unknow | 5 (1.4%) | 4 (2.2%) | 9 (1.6%) | 0.524 |

**Table S1. Analysis of clinicopathological features of patients with lung adenocarcinoma in Training Cohort and Validation Cohort.**
